# Clinical characteristics of epilepsy in resource‐limited communities in Punjab, Northwest India

**DOI:** 10.1002/epi4.12439

**Published:** 2020-11-01

**Authors:** Gagandeep Singh, Sachi Singhal, Suman Sharma, Birinder S. Paul, Namita Bansal, Anurag Chaudhary, Sarit Sharma, Rajnder K. Bansal, Jatinder S. Goraya, Raj K. Setia, Josemir W. Sander

**Affiliations:** ^1^ Research & Development Unit Dayanand Medical College Ludhiana India; ^2^ Department of Neurology Dayanand Medical College Ludhiana India; ^3^ NIHR University College London Hospitals Biomedical Research Centre UCL Queen Square Institute of Neurology London UK; ^4^ Department of Social & Preventive Medicine Dayanand Medical College Ludhiana India; ^5^ Department of Paediatrics Dayanand Medical College Ludhiana India; ^6^ Punjab Remote Sensing Centre Ludhiana India; ^7^ Chalfont Centre for Epilepsy Chalfont St Peter UK; ^8^ Stichting Epilepsie Instellingen Nederland (SEIN) Heemstede The Netherlands

**Keywords:** classification, epileptic syndromes, etiology, perinatal accidents, prevention

## Abstract

**Objectives:**

To describe clinical characteristics of a community‐based epilepsy cohort from resource‐limited communities in Punjab, Northwest India.

**Methods:**

The cohort was gathered following a two‐stage screening survey. We cross‐sectionally examined and followed up the cohort for one year. A panel of neurologists assigned seizure types, syndromes, and putative etiologies and categorized drug responsiveness.

**Results:**

The cohort of 240 included 161 (67.1%) men, 109 (45.4%) illiterates and 149 (62.1%) unemployed. Current age was >18 years in 155 (64.6%) but age at epilepsy onset was <18 years in 173 (72.1%). Epilepsies due to structural and metabolic causes were diagnosed in 99 (41.3%), but syndromic assignments were not possible in 97 (40.4%). After one year, drug‐resistant epilepsy was established in 74 (30.8%). Perinatal events (n = 35; 14.6%) followed by CNS infections (n = 32; 13.3%) and traumatic brain injury (n = 12; 5.0%) were common risk factors. Most of those with CNS infections (n = 19; 63.3%), perinatal antecedents (n = 23; 76.7%), and other acquired risk factors (n = 27; 90.0%) presented with epilepsy due to structural and metabolic causes. Perinatal events were the putative etiology for nearly 40.7% of generalized epilepsies due to structural and metabolic causes and 28.2% of all epilepsies with onset <10 years.

**Significance:**

Existing classifications schemes should be better suited to field conditions in resource‐limited communities in low‐ and middle‐income countries. The finding of drug‐resistant epilepsy in nearly at least a third in a community‐based sample underscores an unmet need for enhancing services for this segment within healthcare systems. Perinatal events, CNS infections, and head injury account for a third of all epilepsies and hence preventative interventions focusing on these epilepsy risk factors should be stepped up.

**Key points**

- Little is known about the clinical characteristics of epilepsy in resource‐limited settings in the South Asia.
- Perinatal adverse events, CNS infections, and traumatic brain injury are risk factors in nearly one‐third of epilepsies in community samples.
- Perinatal adverse events, CNS infections, and traumatic brain injury are preventable risk factors for epilepsy.
- At least one‐third of all epilepsies in community‐based samples might be drug‐resistant.

## 1 INTRODUCTION

The WHO South‐East Asian Region is home to nearly 30 million people with epilepsy, representing more than half of the world's epilepsy burden.^1^, ^2^ Countries in this region shoulder high disease burdens also because of the sheer enormity of untreated epilepsy and excess of premature mortality associated with it.^3^, ^4^ The characteristics, patterns, and outcomes of epilepsy in South Asia are distinctive due to differing risk factors in comparison with western countries, high treatment gaps, and resource limitations.^5^, ^6^ The Commission of Asian and Oceanian Affairs of the International League Against Epilepsy (ILAE) recognizes the understanding of the causes of epilepsy in the Asian‐Oceanian region as a research priority.^2^ Studies from selected regions within Africa, South America, and China have described clinical, electroencephalographic and imaging features, and attributable causes of epilepsy in community‐based samples.^7^, ^8^, ^9^, ^10^ A systematic review could, however, find only a few similar studies from South/east Asia.^11^ Most available reports are hospital‐based, and hence susceptible to referral and selection biases.^12^ Specifically, from across India, these have been reports of prevalence and treatment gap in different communities, albeit small.^13^ Some more recent reports have only partially addressed clinical characteristics.^14^, ^15^ None seem to have used the most current International League Against Epilepsy (ILAE) operational definitions, classifications and criteria applicable to seizures, epilepsy and (antiseizure) drug resistance.^16^, ^17^, ^18^, ^19^ We describe the clinical characteristics of epilepsy using current classification and criteria in a representative community‐based sample from communities with limited resources in the Punjab, Northwest India.

## 2 METHODS

**FIGURE 1:**
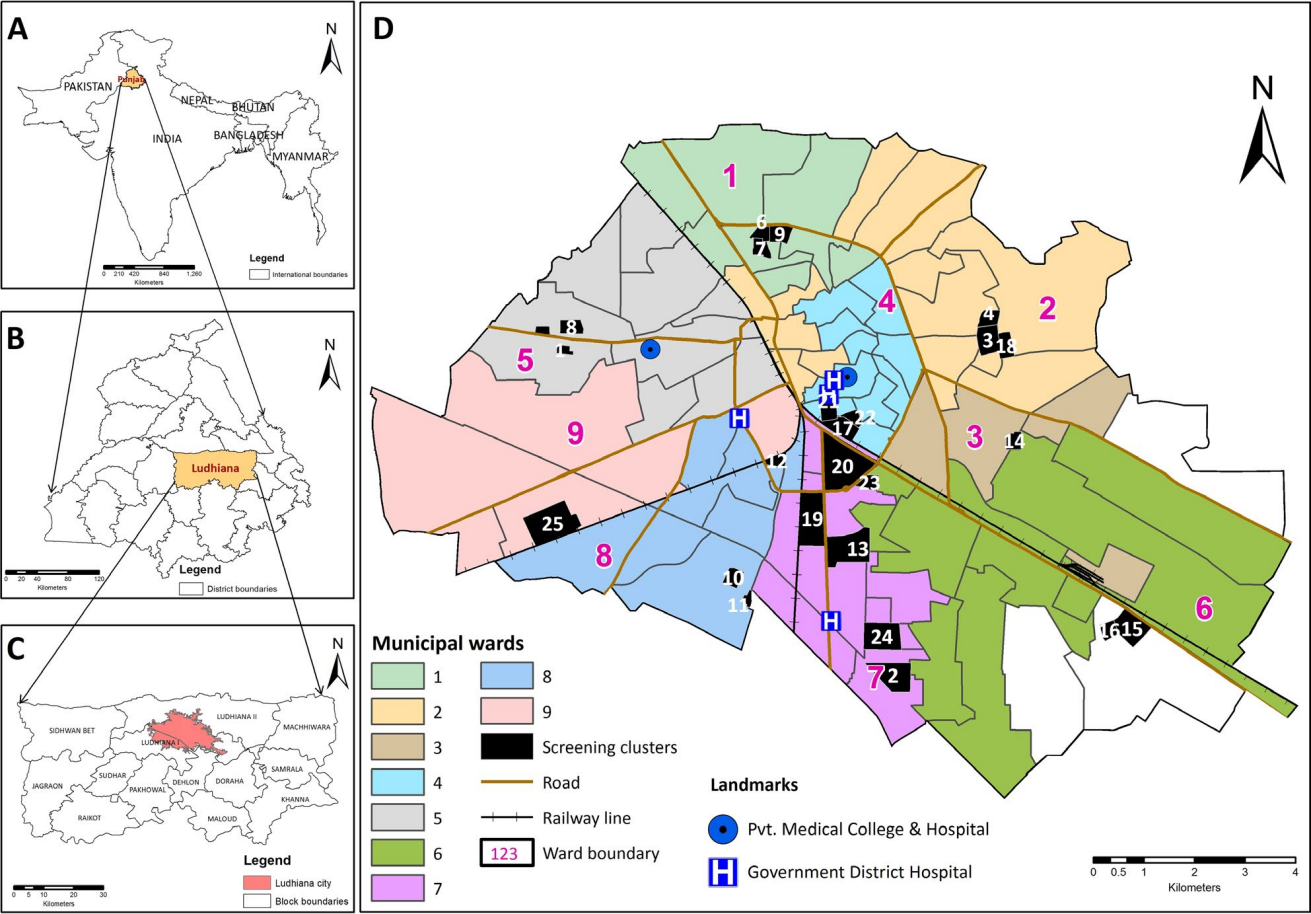
Study area location and map

We cross‐sectionally assessed a sample of people with epilepsy and then followed them up for one year to characterize their clinical, imaging, and electroencephalographic (EEG) characteristics as well as drug responsiveness and outcomes. People were recruited to a cluster‐randomized trial of home‐based care following a community‐based, two‐step survey (described elsewhere^20^) during which 59 509 people were screened for epilepsy in urban and periurban rural areas of Ludhiana in the Northwest Indian state of Punjab. Briefly, purpose‐trained field workers carried out a door‐to‐door screening in 24 clusters of around 2000 people each (Figure 1) using a previously validated questionnaire between May 01, 2017, and June 22, 2018. Screen‐positive people were then invited for evaluation by neurologists (including a pediatric neurologist) specialized in epilepsy at a tertiary‐care hospital facility between May 23, 2017, and July 01, 2018 (Table S1). During this evaluation, they had a standard 1‐ to 2‐hour awake and sleep (if possible) EEG recording (24 channel, Xltek, Ontario, Canada) and MRI brain scan using an epilepsy‐appropriate protocol on a 3T scanner (Skyra, Siemens, Munich, Germany; 20 channels for head coil). Sequences in the scanning protocol included a T1‐weighted 3D‐volumetric acquisition, in addition to T1 and T2 oblique coronals and susceptibility weighted images.

### 2.1 Clinical characteristics

Three neurologists jointly reviewed case notes. They confirmed diagnoses, elucidated semiology, and established seizure types, syndromes, and etiologies by consensus using standard terminology and the current conceptual and operational (2014) definitions of epilepsy.^16^, ^17^ The basic and advanced versions of the 2017 ILAE epilepsy syndromic classifications were used.^18^ Attributable causes were likewise formulated according to a simplified and a more detailed scheme using structured forms.^21^ The simplified scheme encompassed adverse perinatal events, CNS infections (neurocysticercosis included), other acquired cerebral insults (stroke, TBI, and tumors), presumed genetic generalized, and self‐limited focal epilepsies and miscellaneous conditions. Perinatal disorders were considered to be causal, when interrogations disclosed premature birth, unattended (at home) delivery, prolonged labor, instrumental, or operative delivery with postnatal encephalopathy and/or poor feeding and excessive crying.^22^ Neurocysticercosis was diagnosed and further classified into solitary active, solitary inactive, and multiple (mixed stage) forms according to recent consensus criteria.^23^

### 2.2 Follow‐up

Participants were initiated (or continued, if already on appropriate treatment) on antiseizure medications (ASMs) when indicated using a pragmatic approach. They were followed up monthly either at a stand‐alone clinic at the Government District Hospital or during home visits by a study nurse who could schedule interim consultations with the study neurologists at hospital for the participants in the home‐based care group. Participants attending clinic were allowed unscheduled visits, and all could make emergency visits as required. A different researcher recorded seizure control, medication adherence, and adverse effects in the previous month during separate home visits. All participants received ASMs free of charge on a monthly basis.

Previously used ASMs, currently used, and during one year of follow‐up, maximal doses, formulations, seizure control, and adverse effects during their use and reasons for discontinuation (if applicable) were recorded. Previous use of ASMs was recorded at initial evaluation and subsequent use recorded prospectively. We stratified seizure outcome following each ASM used into three groups in keeping with current ILAE criteria for DRE.^19^ These were as follows:

(1) Complete seizure freedom with or without adverse effects over three times the pretrial interseizure interval or 12 months, whichever was longer,

(2) Treatment failure (occurrence of seizures during the specified interval above) with or without adverse effects,

(3) Undetermined outcome when the observation period was less than three times the longest pretrial interseizure interval.

Subsequently, the ILAE definition of drug‐resistant epilepsy was applied to categorize epilepsy as drug‐resistant, drug‐responsive, or indeterminate.

Once a participant completed a year of follow‐up, neurologists by consensus reassigned seizure, syndrome, and attributable causes, and entered into structured forms.

### 2.3 Comorbidities

We adapted complains’ inventories from an epilepsy‐specific somatic comorbidity index and from a Canadian tool for psychiatric comorbidities to record somatic and psychiatric comorbidities during follow‐up.^24^, ^25^ Spontaneous medical and psychiatric complaints during follow‐up were also noted. Formal medical or psychiatric and psychometric assessments were requested to deal with problems arising. Medical and psychiatric comorbidities were inferred from review of the case notes and matched to the inventories.

In women with epilepsy in the reproductive age group (18‐45 years), folic acid and contraceptive method use during the follow‐up period were noted. The occurrence of pregnancies, terminations, and outcome of each pregnancy over one year were recorded.

### 2.4 Sociodemographic categorization

During the initial evaluations, we ascertained household income, education, and employment to assign socioeconomic status according to the Kuppuswamy scale (revised, 2015 version). ^26^ This validated scale, widely used in India, assigns deprivation level to one of five ordinal classes.

### 2.5 Statistical analysis

We used descriptive statistics to analyze sociodemographic variables and the proportions of various seizure types, epilepsy syndromes, etiological risk factors, drug resistance, and comorbidities. To establish associations between various syndromic diagnoses and attributable causes and various sociodemographic variables, we used univariate analyses (with the chi‐square test) and multinomial regression. For both analyses, we consigned presumed genetic generalized epilepsies as the reference category. We obtained relative risk ratios by exponentiating the linear equations generated and regression coefficients for unitary changes in the predictor variables estimated. Lastly, Venn diagrams created to explore the relationship between different seizure types, syndromes, and etiologies. Stata ver. 15 (StataCorp, TX, USA) was used for the analyses.

The study was scrutinized and approved by the Institutional Ethics Committee of Dayanand Medical College & Hospital, Ludhiana, India (IEC no. 2017‐281) and registered with the Clinical Trial Registry of India (Re.: 2017/09/015380). Participants provided written informed consent and parents or guardian provided assent for minors or those not having capacity.

## 3 RESULTS

We recruited 240 individuals between 1 and 80 years of age and of whom, 161 (67.1%) were males. EEGs were recorded in 237, and an MRI was performed in 200. At enrollment, 230 (95.8%) had a net family income of less than US $ 250/month, 149 (62.1%) were unemployed with 43 (17.9%) on daily wages, and 152 (63.3%) were either illiterate or had barely completed primary school (Table 1). Thus, 194 (80.8%) were in the lower segment on the Kuppuswamy scale.^26^

**TABLE 1:** Sociodemographic features of the cohort

Sociodemographic features	Number^a^(%) n = 240	<18 y age of onset (%) n = 173	Females (%) n = 79
Gender			
Female	79 (32.9%)	60 (34.7%)	
Religion			
Hindus	138 (57.5%)	98 (56.6%)	49 (62.0%)
Sikhs	94 (39.2%)	70 (40.5%)	25 (31.6%)
Others	8 (3.3%)	5 (2.9%)	5 (6.3%)
Ethnic origin			
Local	150 (62.5%)	111 (64.2%)	51 (64.6%)
Migrants	90 (37.5%)	62 (35.8%)	28 (35.4%)
Education			
Illiterate	109 (45.4%)	90 (52.0%)	39 (49.4%)
Below high school	152 (63.3%)	116 (67%)	54 (68%)
High school and above	88 (36.7%)	57 (32.9%)	25 (31.6%)
Occupation–Unemployed	149 (62.1%)	125 (72.3%)	71 (89.9%)
Family income < US $ 250/month	230 (95.8%)	167 (96.5%)	76 (96.2%)
Social class^b^‐ Lower	194 (80.8%)	139 (80.3%)	67 (84.8%)
Marital status–Married	89 (37.1%)	37 (21.4%)	35 (44.3%)
Habitat			
Rural	37 (14.6%)	28 (16.2%)	13 (176.5%)
Urban	203 (84.8%)	142 (82.1%)	66 (83.5%)

^a^ Total number of subjects analyzed = 240.

^b^ Social class designated according to Kuppuswamy scale (see text) (Ref. 22).

Epilepsy duration varied from 1 to 62 years (median: 11 years; 95% confidence intervals, 5‐21 years). Over half of the participants (53.3%) had had seizures for more than 10 years. Age at onset was <5 years in 75 (31.3%), <10 years in 108 (45.0%), and less than 18 years in 173 (72.1%) people. Age at neurological evaluation was over 18 years in 155 (64.6%) people. Prior to the neurological evaluation, 116 (48.3%) reported at least one seizure in a month.

There were 189 (78.8%) participants with generalized tonic‐clonic seizures and 52 (21.7%) with focal seizures with or without impaired awareness (Table 2). Seventy‐four had more than one seizure type (30.8%). We diagnosed focal epilepsies due to structural and metabolic causes in 97 (40.4%) people, presumed genetic generalized epilepsies in 37 (15.4%), and generalized epilepsies due to structural/metabolic disorders in 18 (7.5%). Of the 97 people with focal epilepsy due to structural and metabolic causes, 47 (48.5%) reported focal seizures and 16 (16.5%) focal and generalized seizures. Among acquired epilepsies, perinatal insults were the commonest (n = 35; 14.6%), followed by neurocysticercosis (n = 28; 11.7%) and TBI (n = 12:5.0%; Table 2). The proportion with a perinatal event increased to 19.1% (33 out of 173) in those with age of onset <18 years and to 25.9% (28 out of 108) with onset <10 years.

**TABLE 2:** Seizure types, epilepsy syndromes, and etiologies

ILAE seizure types	
Generalized tonic‐clonic	189 (78.8%)
Focal seizures with temporal lobe automatisms and awareness impaired	24 (10.0%)
Focal with elementary clonic seizures	14 (5.8%)
Focal with elementary sensory seizures	12 (5.0%)
Focal with experiential sensory seizures	2 (0.8%)
Focal asymmetric tonic seizures	6 (2.5%)
Focal tonic seizures	4 (1.7%)
Versive seizures	2 (0.8%)
Myoclonic seizures	20 (8.3%)
Absence seizures	1 (0.4%)
Spasms	2 (0.8%)
Indeterminate seizure types	32 (13.3%)
ILAE epilepsy syndromes	
Focal epilepsies due to structural and metabolic causes	97 (40.4%)
Limbic epilepsies mesial temporal epilepsy with hippocampal sclerosis	18 (7.5%)
HHE syndrome	4 (1.7%)
Presumed Rasmussen's syndrome	1 (0.4%)
Epilepsy with continuous spike waves during sleep	1 (0.4%)
Presumed genetic generalized epilepsy	37 (15.4%)
	12 (5.0%)
Generalized epilepsy due to structural and metabolic causes West syndrome	14 (5.8%)
Indeterminate	82 (34.2%)
Aetiologies	
Perinatal hypoxia	35 (14.6%)
Neonatal hypoglycemia	2 (0.8%)
CNS infections	32 (13.3%)
Neurocysticercosis	28 (11.7%)
Postmeningitis epilepsy	4 (1.7%)
Other acquired epilepsy risk factors	27 (11.3%)
Traumatic brain injury	12 (5%)
Stroke	3 (1.3%)
Tumor	1 (0.4%)
Hippocampal sclerosis	9 (3.8%)
Cortical developmental malformations	4 (1.7%)
Presumed genetic epilepsies	39 (16.3%)
Generalized	37 (15.4%)
Focal	2 (0.8%)
Miscellaneous	5 (2.1%)
Down's syndrome	2 (0.8%)
Alcohol and drug abuse	3 (1.3%)
Indeterminate	97 (40.4%)

**FIGURE 2:**
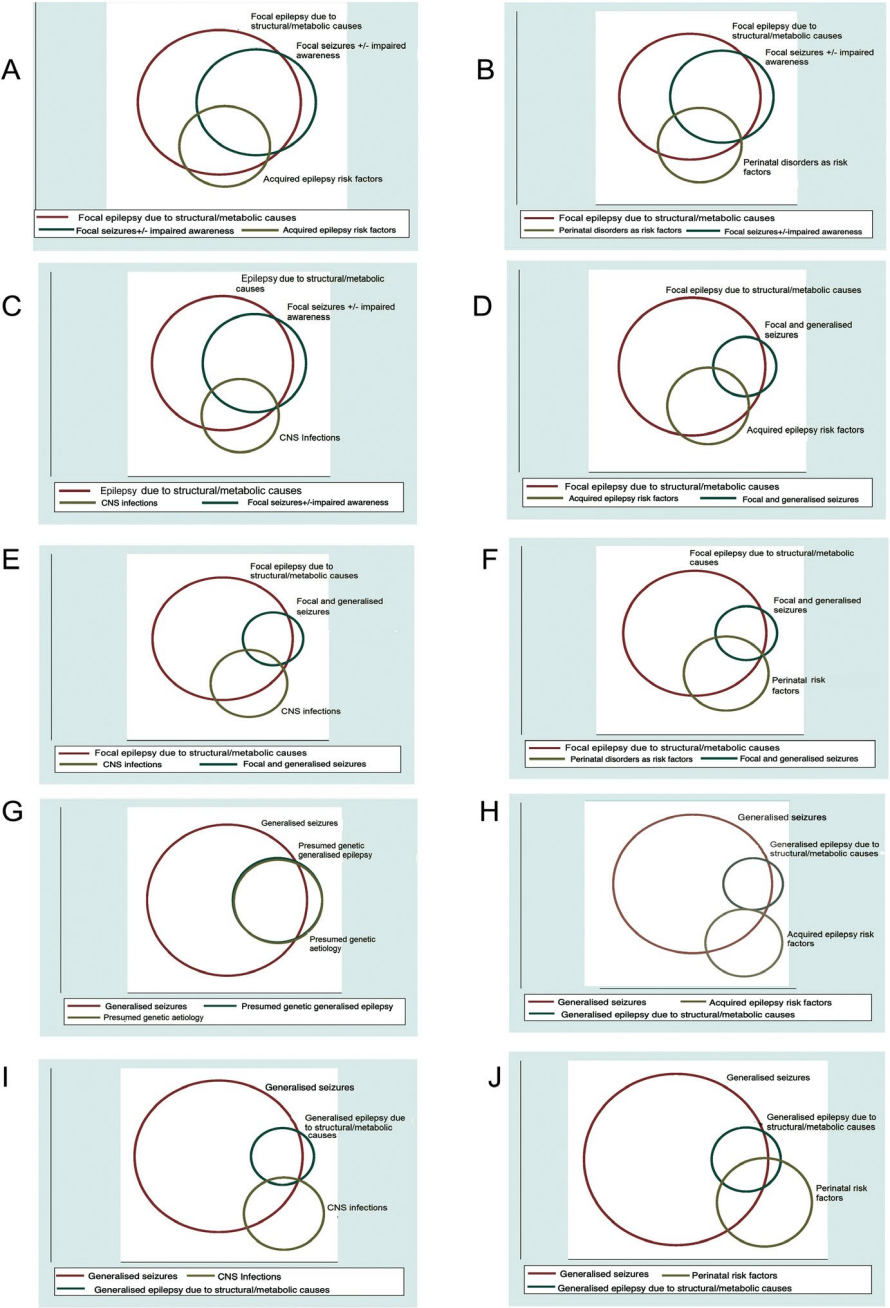
Venn diagrams depicting the relationships between seizure types, epilepsy syndromes, and putative etiologies, for example, between symptomatic/probably symptomatic focal epilepsies, focal seizures with or without impaired awareness and (A) acquired epilepsy risk factors (stroke, tumor, and traumatic brain injury), (B) perinatal risk factors, and (C) CNS infections; between focal epilepsies due to structural and metabolic causes, both focal and generalized seizures and (D) acquired epilepsy risk factors, (E) perinatal risk factors, and (F) CNS infections; between generalized seizures and (G) genetic generalized epilepsies and between generalized seizures, epilepsy due to structural and metabolic causes and (H) acquired epilepsy risk factors, (I) CNS infections, and (J) perinatal risk factors

Putative etiologies for focal epilepsies due to structural and metabolic causes included CNS infections (n = 29; 29.9%), perinatal risk factors (n = 23; 23.7%), and other acquired epilepsy risk factors (n = 27; 27.8%). In those with focal seizures (n = 47), epilepsy could be attributed to acquired risk factors in 13 (27.7%), to perinatal risk factors in 11 (23.4%), and to CNS infections in 9 (19.2%) (Figure 2A‐C). The probabilities in someone with both focal and generalized seizures and a syndromic diagnosis of epilepsy due to structural and metabolic causes (n = 16) could be attributed to acquired risk factors were 18.8%, to perinatal risk factors were 31.3%, and to CNS infections 12.5% (Figure 2D‐F). Most participants (n = 34; 97.1%) with genetic generalized epilepsy had generalized seizures (Figure 2G), and in comparison only one (6.3%) of those with prior CNS infections (Figure 2I) and six (37.5%) with perinatal insults (Figure 2J) had generalized seizures. Of those with generalized seizures (n = 128; 53.3%), 37 (29%) were considered to be having presumed genetic generalized epilepsies and 16 (12.5%) generalized epilepsy due to structural/metabolic causes. Among those with a syndromic diagnosis of generalized epilepsy due to structural/metabolic causes (n = 16), perinatal risk factors were identified in seven (44%) but in none and only one, respectively, were other acquired epilepsy risk factors and CNS infections identified.

Neurological comorbidities included global developmental delay in 32 (13.3%), migraines in 14 (5.8%), focal neurological deficits in 8 (3.4%), stroke in 5 (2.1%), and sleep disturbances in 4 (1.7%). Psychiatric comorbidities included alcohol and substance abuse (n = 22; 9.1%), mood disorders (n = 20; 8.3%), anxiety (n = 5; 2.1%), and significant memory complaints (n = 13; 5.4%). Severe intellectual disability was noted in nine (3.8%), and attention deficit hyperactive disorder in six (2.5%). Concomitant psychogenic nonepileptic attacks were ascertained in nine (3.8%).

### 3.1 EEG and MR findings

**FIGURE 3:**
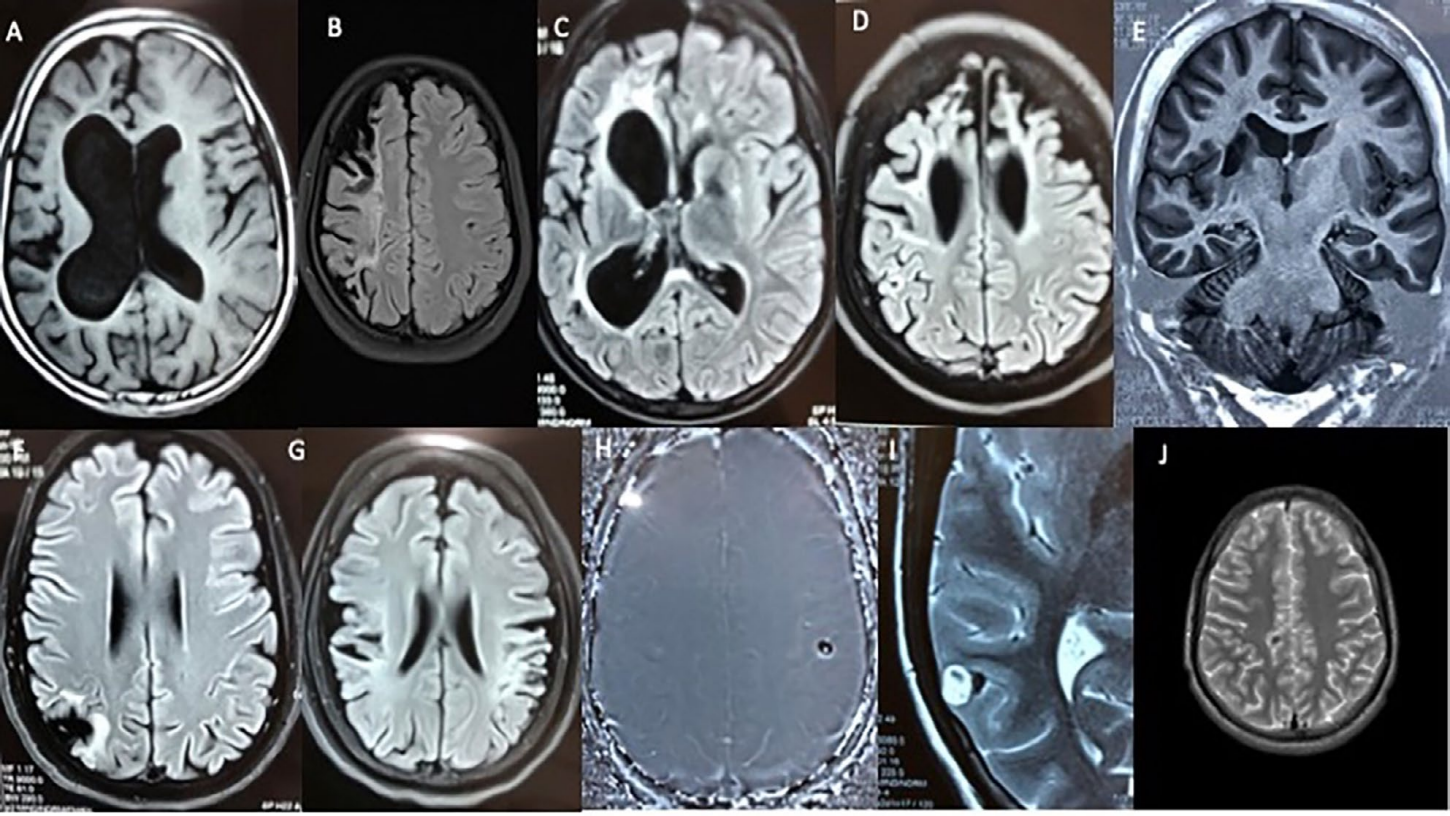
Representative spectrum of magnetic resonance imaging findings in the cohort: A, T1‐weighted image showing right hemisphere cortical and subcortical and periventricular (*ex‐vacuo* dilatation of the right lateral ventricle) atrophy in an individual with HHE syndrome; B, fluid attenuated inversion recovery (FLAIR) image with more focal gyral atrophy with subjacent white matter hyperintensities restricted to the right frontal lobe in an individual with apparent generalized tonic‐clonic seizures and a syndromic diagnosis of focal epilepsy following a presumed perinatal event; C, FLAIR image with predominant subcortical and periventricular atrophy and overlying white matter hyperintensities in another individual with HHE syndrome; D, FLAIR image with gyral atrophy, gliosis, subjacent subcortical hyperintensities, and *ex‐vacuo* dilatation of the frontal horns of the lateral ventricles in a child with apparent generalized tonic seizures and generalized epilepsy again resulting from a presumed perinatal event; E, T1‐weighted oblique coronal inversion recovery image showing right hippocampal atrophy and cystic encephalomalacia in the right basal ganglia in an individual with right mesial temporal lobe epilepsy; F, FLAIR image with focal encephalomalacia and underlying subcortical hyperintensities in the right parietal lobe in an individual with focal (post‐traumatic) epilepsy; G, FLAIR image showing bilateral gyral atrophy with subjacent hyperintensities involving the temporo‐parietal opercula underlying symptomatic multifocal epilepsy again associated with a perinatal event; H, phase contrast image depicting a calcified lesion with an eccentric scolex presumably, the calcified stage of a parenchymal cysticercus cyst in someone with symptomatic focal epilepsy; I, T2 image with a cysticercus in the granulo‐nodular stage in the right posterior temporal lobe; and J, T2 image with a minute calcified lesion, presumably the calcified stage of a parenchymal cysticercus

A presumed genetic generalized epilepsy pattern (n = 34; 14.2%) was the most common finding followed by focal epileptiform activity from the extra‐ and lateral‐temporal (n = 30; 12.5%) and antero‐mesial temporal regions (n = 26; 10.8%) on EEG (Table S2). The three most common imaging findings included lesions compatible with a diagnosis of neurocysticercosis (n = 28; 11.7%), focal/regional encephalomalacia or gliosis (n = 28; 11.7%), and mesial temporal sclerosis (n = 9; 3.7%; Table S2). Some representative imaging findings are displayed in Figure 3A‐H.

**TABLE 3:** Comparative characteristics of drug‐responsive and drug‐resistant epilepsies in the cohort

Characteristics	Drug responsiveness			*P*‐value*
	Drug‐responsive epilepsy (n = 94)	Drug‐resistant epilepsy (n = 74)	Indeterminate (n = 72)	
Epilepsy onset age bands (years)				
<10	30 (31.9%)	44 (59.5%)	34 (47.2%)	.007*
10‐20.0	38 (40.4%)	20 (27.0%)	19 (26.4%)	
21‐30	13 (13.8%)	6 (8.1%)	9 (12.5%)	
31‐40	6 (6.4%)	3 (4.1%)	8 (11.1%)	
>40	7 (7.4%)	1 (1.4%)	2 (2.8%)	
Epilepsy onset (Mean ± SD) (years)	17 ± 15	10 ± 10	14 ± 12	<.0001*
Duration of epilepsy (Mean ± SD) (years)	13 ± 11	15 ± 13	14 ± 9	.171
Current age (Mean ± SD) (years)	28 ± 17	24 ± 15	26 ± 14	.11
Gender: Female (%)	27 (28.7%)	27 (36.5%)	25 (34.7%)	.29
Religion				
Hindu	54 (57.4%)	48 (64.9%)	37 (51.4%)	.56
Sikh	38 (40.4%)	24 (32.4%)	32 (44.4%)	
Other	2 (2.1%)	2 (2.7%)	3 (4.2%)	
Ethnic origin				
Migrant	35 (37.2%)	26 (35.1%)	29 (40.3%)	.78
Local	59 (62.8%)	48 (64.9%)	43 (59.7%)	
Education				
Illiterate	41 (43.6%)	38 (51.4%)	30 (41.7%)	.32
Literate	53 (56.4%)	36 (48.6%)	42 (58.3%)	
Occupation				
Unemployed	51 (54.3%)	54 (73.0%)	44 (61.1%)	.01*
Employed	43 (45.7%)	20 (27.0%)	28 (38.9%)	
Family income (INR)				
<18 000	89 (94.7%)	71 (95.9%)	70 (97.2%)	.7
>18 000	5 (5.3%)	3 (4.1%)	2 (2.8%)	
Social class				
Lower	74 (78.7%)	62 (83.8%)	58 (80.6%)	.41
Upper	20 (2.3%)	12 (16.2%)	14 (19.4%)	
Marital status				
Married	35 (37.2%)	25 (33.8%)	29 (40.3%)	.64
Single	59 (62.8%)	49 (66.2%)	43 (59.7%)	
Habitat				
Rural	13 (13.8%)	12 (16.2%)	10(13.9%)	.67
Urban	81 (86.2%)	62 (83.8%)	62 (86.1%)	
Epilepsy syndrome				
Genetic generalized epilepsies	18 (19.1%)	10 (13.5%)	12 (16.7%)	.33
Generalized epilepsies due to structural and metabolic causes	5 (5.3%)	10 (13.5%)	3 (4.2%)	.06
Focal epilepsies due to structural and metabolic causes	35 (37.2%)	34 (45.9%)	28 (38.9%)	.12
Seizures not necessarily requiring a diagnosis of epilepsy/Indeterminate syndrome	36 (38.3%)	20 (27.0%)	29 (40.3%)	.12
Etiology				
Genetic/presumed genetic causes	18 (19.1%)	9 (12.2%)	12 (16.7%)	.22
Perinatal events (including anoxic/ischemic disorders around birth)	10 (10.6%)	15 (20.3%)	9 (12.5%)	.08*
CNS infections	11 (11.7%)	7 (9.5%)	11 (15.3%)	.64
Other acquired epilepsy risk factors	14 (14.9%)	9 (12.2%)	7 (9.7%)	.61
Miscellaneous/Indeterminate etiologies	41 (43.6%)	34 (45.9%)	33 (45.8%)	.76
Somatic comorbidities				
Acute infection episodes	14 (14.9%)	17 (23.0%)	5 (6.9%)	.18
Tuberculosis	0(0%)	2 (2.7%)	0 (0%)	.11
Protein energy malnutrition	0(0%)	1 (1.4%)	0 (0%)	.26
Fracture	3 (3.2%)	0 (0%)	0 (0%)	.12
Others	11 (11.7%)	12 (16.2%)	15 (20.8%)	.43
Neurological disorders				
Migraine	8 (8.5%)	4 (5.4%)	2 (2.8%)	.44
Cerebrovascular Disease	2 (2.1%)	2 (2.7%)	2 (2.8%)	.81
Other focal neurological deficits	3 (3.2%)	3 (4.1%)	2 (2.8%)	.77
Global developmental delay	9 (9.6%)	15 (20.3%)	8 (1.1%)	.05*
Mental challenge	4 (4.3%)	4 (5.4%)	1 (1.4%)	.73
Attention Disorder & Hyperactive Syndrome	3 (3.2%)	4(5.4%)	3 (4.24%)	.48
Sleep disturbances	2 (2.1%)	1 (1.4%)	1 (1.4%)	.71
Psychiatric comorbidities				
Depression	6 (6.4%)	10 (13.5%)	4 (5.6%)	.12
Suicidal ideation	3 (3.2%)	4 (5.4%)	1 (1.4%)	.47
Anxiety	1 (1.1%)	4 (5%)	1 (1.4%)	.21
Obsessive Compulsive Disorder	1 (1.1%)	0(0%)	0 (0%)	.37
Concomitant psychogenic nonepileptic attacks	0 (0%)	8 (10.8%)	1 (1.4%)	.001*
Alcohol and substance abuse	13 (13.8%)	2 (2.7%)	7 (9.7%)	.01*
Abnormal EEG	44 (46.8%)	44 (59.5%)	35 (48.6%)	.12
Abnormal MRI	38 (48.1%)	27 (44.3%)	30 (41.7%)	.65

* *P* values represent probabilities based on comparison of the drug‐responsive and drug‐resistant groups.

### 3.2 Drug responsiveness

After one‐year of follow‐up, 74 (30.8%) participants fulfilled ILAE criteria for DRE.^19^ Drug responsiveness could not be established in 72 (30.0%) due to an inadequate observation period. People with DRE were younger (mean age: 10 ± 10 years; *P* < .0001), more often developmentally delayed on a global scale (n = 15 out of 74; 20.3%; *P* = .05), more likely to have concomitant psychogenic nonepileptic attacks (n = 8 out of 74; 10.8%; *P* = .001), less likely to use alcohol and other recreational substances (n = 2 out of 74; 2.7%; *P* = .01), and less likely to be employed (n = 20; [27.0%] out of 74 compared to 43 [45.7%] of 94 with DRE; *P* = .01) in comparison with their drug‐responsive counterparts (Table 3). Besides, a trend for greater frequency of generalized epilepsy due to structural and metabolic causes (10 [27%] out of 74 compared to 5 [5.3%] of 94; *P* = .06) and perinatal events as risk factors (15 [24%] out of 74 compared to 10 [10.6%] of 94; *P* = .08) was noted in the DRE subgroup.

### 3.3 Sociodemographic determinants of syndromic and etiological diagnoses

A syndromic assignment of presumed genetic generalized epilepsy was associated with the age band, 11‐20 years (using the age band 1‐10 years as reference) in univariate analysis (OR: 2.90; 95% CI, 1.25‐6.71; *P* = .01). A negative association was found between all epilepsies due to structural and metabolic causes and the age band, 21‐30 years (in comparison with the reference band, 1‐10 years) (*P* = .05; OR: 0.36; 95%CI, 0.13‐1.00). Acquired epilepsy risk factors were most common in the age band, over 40 years (*P* = .001; OR: 15.97; 95%CI, 3.14‐81.30). In multinomial analyses, presumed genetic epilepsies were associated with age of onset (*P* = .03; RR: 1.04; 95%CI, 1.00‐1.08) and participants with acquired risk factors were older in comparison with the reference group of presumed genetic epilepsies (*P* = .005; RR: 1.06; 95%CI, 1.02‐1.12). People with focal epilepsies due to structural and metabolic causes were less likely to be employed (*P* = .041; RR: 0.34; 95%CI, 0.13‐0.96), and those with perinatal events were less likely to have undergone any schooling (*P* = .024; RR: 0.20; 95%CI, 0.05‐0.81).

## 4 DISCUSSION

The spectrum of seizures, syndromes, and attributable causes with imaging and EEG findings in community‐based cohorts from South/South‐East Asian Region have been sparingly described previously.^15^, ^27^, ^28^ Here, we reflect on some distinctive findings in our sample.

The age of epilepsy onset in our cohort was consistent with other reports from other resource‐limited settings. For instance, over half of Kenyan and Honduran population‐based cohorts had onsets before age, 10 years.^7^, ^29^ In comparison, less than one‐third of a prevalent sample from Rochester, Minnesota, USA, dating back to 1980s had onset < 9 years of age.^30^ Epilepsy duration in our cohort was similar to Rochester, of whom over half had epilepsy for more than 10 years.^30^ Predictably, nearly two‐thirds in our cohort were over 18 years of age. The gender bias in our sample with women representing only a third merits discussion. In Honduras, the survey found higher prevalence in women, and in Kenya, women constituted a little over half in a representative sample of people with epilepsy.^7^, ^8^, ^29^ When we examined the segment of screen‐positive people not attending neurological evaluation, the proportion of women was higher but this only partly explained the underrepresentation of women in our final sample. In many communities with limited resources in South‐East Asia, women are not able to travel and this could preclude some of them to attend the hospital for evaluation.^31^ Stigma associated with disclosure widespread in the region might also have precluded attendance or even disclosure during the screening campaign.^32^

The higher proportion of generalized seizures in comparison with focal seizures in our sample is at variance from other studies.^8^, ^33^ Some of the generalized seizures might have in reality been focal seizures evolving to bilateral tonic‐clonic seizures. An under‐reporting of focal seizures might also have been due to cultural issues. Even so, the finding underscores the difficulties in classifying seizures in population‐based settings. Problems associated with assigning syndromes in population‐based samples are well known.^34^, ^35^ The complex relationship, however, between seizure types, epilepsy syndromes, and attributable causes is illustrated by the Venn diagrams (Figure 2A‐H).

Adverse perinatal events were the commonest risk factor in our cohort and higher than in some recent studies from sub‐Saharan Africa. Several settings including premature births, unattended home deliveries, cesarean sections followed by postnatal events such as hypoglycemia in institutional care, all more frequent in resource‐limited communities have been associated with epilepsies.^7^, ^36^, ^37^, ^38^ The estimated median fraction of epilepsies attributed to adverse perinatal events is roughly 11%.^39^ Epilepsies following perinatal insults are frequently associated with comorbidities including motor deficits, mental challenge, and poor scholastic performance.^22^, ^36^, ^40^ In our sample, individuals with an adverse perinatal event as the putative etiology of epilepsy had significantly lower educational grades and a trend toward drug resistance (Table 3). Adverse perinatal events were also more common in younger people. This is consistent with findings from previous studies of prevalence samples of epilepsies worldwide.^7^, ^30^ A lesser frequency in adults could mean that either many died prematurely or less likely, moved into remission. Nonetheless, this suggests the need to contextualize antenatal and perinatal care in resource‐limited communities in LMICs. The lack of skilled birth attendants, poor capacity of health systems, and access issues such as cultural beliefs, distance, and financial limitations are barriers to quality maternal and childcare, much required for improving perinatal outcomes.

The proportion of pharmacoresistance in our sample appears high; however, this might still be an underestimate, as the categorization into DRE or not was not possible in nearly a third of the cases. ^41^ Not many reports of proportion of DRE in population‐based samples of active epilepsy are available. An Italian study found DRE in less than a fifth of people with active epilepsy using older criteria. ^42^ An Egyptian study reported uncontrolled epilepsy, defined as at least a monthly seizure frequency during the previous six months, in about a third of those with active epilepsy. ^43^ This was after selecting out those with pseudointractability due to inappropriate drug choice or doses or due to incorrect diagnosis. Rather our findings align with a Kenyan study reporting seizures on at least a monthly basis in 58% of people. ^8^ We surmise that the high proportion of pharmacoresistance might be due to engaging a prevalent sample, paucity of resources, and access to appropriate care, that is, surgical treatment in impoverished communities. Regardless of the reason, DRE is associated with substantially higher disease burden in comparison with controlled epilepsy and justifies the need to scale up provisions for tackling it in healthcare systems. ^3^, ^4^

Caution should be exercised in the interpreting our findings due to some limitations. Firstly, we included less than half of those who screened positive at the survey stage. Many did not attend the evaluation following screening. The characteristics and outcome of this group of people are described elsewhere. ^44^ Secondly, some potential participants may have been missed during screening due to fear of the consequences of disclosure. Thirdly, most came from a low SES category. This, however, does not mean that the communities from which our sample was drawn were comparably impoverished as we did not collect or have access to general population data. Enlistment of somatic and psychiatric comorbidities was based on spontaneous reporting rather than a structured inquiry, and this may have led to an underenumeration of the conditions. Not least, the classification of epilepsies and attributable causes often remained inadequate, emphasizing the challenges we faced, and hence an urgent need for pragmatic classifications suited to resource‐limited community settings.

Adverse perinatal events, CNS infections (including neurocysticercosis), and acquired risk factors (including traumatic brain injury (TBI) and stroke) accounted for over a third of our sample. All these factors are amenable to primary prevention, and it could be argued that third of epilepsies in our sample could have been prevented. ^39^ Population‐based interventions and initiatives to improve obstetric outcomes, eliminate, or eradicate CNS infections (eg, neurocysticercosis) and prevent stroke and TBI are warranted to reduce the burden of epilepsy in LMICs. ^45^, ^46^ Currently, a handful of trials to improve obstetrical outcome in LMICs are underway, of which only one, a pragmatic trial of improved intrapartum care is assessing its impact on epilepsy incidence in India.^47^, ^48^ Primary prevention trials and initiatives should likewise target a variety of CNS infections, TBI, and stroke. ^49^, ^50^ Lastly, there is a compelling need for translational research assessing secondary prevention, referring to the interruption of epileptogenesis after the occurrence of adverse perinatal events, CNS infection, stroke, or TBI to reduce the burden of epilepsy.

## CONFLICT OF INTEREST

None of the authors have conflicts of interest to report in relation to this work. We confirm that we have read the Journal’s position on issues involved in ethical publication and affirm that this report is consistent with those guidelines.

## AUTHOR CONTRIBUTIONS

GS and JWS conceptualized the study. All provided substantial intellectual contribution to the study design. GS drafted the manuscript with input from SS who was also responsible for data collection and analysis. SS performed the field data collection and assisted the analysis. RKB, BSP, JSG, and GS performed the neurological evaluations. AC and SS supervised the field study. NB undertook the statistical analysis. RKS performed the geographic information mapping for the field operations. JWS provided critical intellectual input to the analysis and to the revision of the manuscript. GS is the guarantor of the study. All reviewed and approved the submitted manuscript.

## Supporting information

Table S1‐S2
