# Supplementary material for: Clinical characteristics of epilepsy in resource‐limited communities in Punjab, Northwest India
Source: Epilepsia Open. 2020 Nov 1;5(4):582–95. doi: 10.1002/epi4.12439 (PMC7733663; doi:10.1002/epi4.12439)
Supplement: Supplementary file 1 — Table S1‐S2 [file EPI4-5-582-s001.docx]

Supporting Tables

**Clinical Characteristics of Epilepsy in Resource-Limited Settings: A Population Study**

Gagandeep Singh^1,2,3^, Sachi Singhal^1^, Suman Sharma^1^, Birinder S. Paul^2^, Namita Bansal^1^, Anuraag Chowdhury^4^, Sarit Sharma^4^, Rajnder K. Bansal^2^, Jatinder S. Goraya^5^, Raj K. Setia^6^, Josemir W. Sander^3,7,8^

Table S1: Summary of population screened, screen-positives, eligible subjects and refusals.

| **Cluster No.** | **Population screened** | **Epilepsy**  **screen positives** | **Number accepted to study** | **Number found ineligible** | **Number that refused assessment** |
| --- | --- | --- | --- | --- | --- |
| 1 | 4000 | 29 | 10 | 09 | 10 |
| 2 | 2000 | 26 | 10 | 06 | 10 |
| 3 | 4000 | 32 | 10 | 07 | 15 |
| 4 | 3005 | 24 | 10 | 05 | 09 |
| 5 | 2809 | 25 | 10 | 06 | 09 |
| 6 | 2707 | 23 | 10 | 06 | 07 |
| 7 | 2069 | 20 | 10 | 04 | 06 |
| 8 | 2800 | 20 | 10 | 04 | 06 |
| 9 | 2000 | 27 | 10 | 13 | 04 |
| 10 | 2940 | 23 | 10 | 03 | 10 |
| 11 | 2212 | 21 | 10 | 03 | 08 |
| 12 | 2608 | 29 | 10 | 03 | 16 |
| 13 | 2471 | 22 | 10 | 05 | 07 |
| 14 | 2504 | 19 | 10 | 03 | 06 |
| 15 | 2171 | 30 | 10 | 11 | 09 |
| 16 | 2084 | 22 | 10 | 5 | 07 |
| 17 | 2020 | 20 | 10 | 0 | 10 |
| 18 | 2307 | 20 | 10 | 3 | 7 |
| 19 | 2212 | 28 | 10 | 4 | 14 |
| 20 | 2636 | 19 | 10 | 4 | 5 |
| 21 | 2871 | 27 | 10 | 5 | 12 |
| 22 | 2437 | 26 | 10 | 3 | 13 |
| 23 | 1300 | 23 | 10 | 2 | 11 |
| 24 | 1346 | 18 | 10 | 2 | 6 |
| Total | 59,509 | 573 | 240 | 116 | 217 |

Table S2. Spectrum of EEG and MRI findings in the cohort.

| Findings | Number* (%) | < 18 yrs age of onset (%) n=173 |
| --- | --- | --- |
| ***(a)   EEG*** |  |  |
| Presumed genetic generalized epilepsy | 34 (14%) | 28 (12%) |
| BECTS | 2 (1%) | 2 (1%) |
| Focal epileptiform activity | 56 (23%) | 45 (19%) |
|          *Mesial temporal lobe epileptiform activity* | 26 (11%) | 17 (7%) |
| Symptomatic generalized epilepsy | 7 (3%) | 7 (3%) |
| Diffuse encephalopathic pattern | 13 (5%) | 12 (5%) |
| Focal structural abnormalities | 6 (3%) | 4 (2%) |
| Non-specific abnormalities | 5 (2%) | 3 (1%) |
| ***(b)   MRI*** |  |  |
| Focal/regional gliosis/encephalomalacia | 28 (12%) | 22 (9%) |
| Mesial temporal sclerosis | 9 (4%) | 6 (3%) |
| Cortical developmental malformation | 4 (2%) | 4 (2%) |
| Neurocysticercosis | 28 (12%)  6 (3%)  13 (6%)  9 (4%) | 20 (8%) |
|          *Solitary; active* |  |  |
|          *Solitary; inactive (calcified)* |  |  |
|          *Multiple (including mixed stages)* |  |  |
| Chronic cerebral infarct | 4 (2%) | 1 (0.4%) |
| Tumor | 2 (1%) | 2 (1%) |
| Others | 20 (8%) | 19 (8%) |

**Note:** *, Total number of subjects analyzed = 240; EEGs were done in 237 and MRIs in 200.
